# *N*,*N*-Dicyclo­hexyl-3,5-dinitro­benzamide

**DOI:** 10.1107/S1600536812038500

**Published:** 2012-09-19

**Authors:** Sohail Saeed, Naghmana Rashid, Ray J. Butcher, Sema Öztürk Yildirim, Rizwan Hussain

**Affiliations:** a Department of Chemistry, Research Complex, Allama Iqbal Open University, Islamabad 44000, Pakistan; b Department of Chemistry, Howard University, 525 College Street NW, Washington DC 20059, USA; c Department of Physics, Faculty of Sciences, Erciyes University, 38039 Kayseri, Turkey; d National Engineering & Scientific Commission, PO Box 2801, Islamabad, Pakistan

## Abstract

In the title compound, C_19_H_25_N_3_O_5_, the benzene ring is not coplanar with the amide group [dihedral angle = 61.90 (5)°]. The cyclo­hexyl rings are in chair conformations. There is a strong inter­molecular inter­action between the C=O group of the amide group and the nitro group of an adjoining mol­ecule, with a short O⋯N distance of 2.7862 (17) Å. In the crystal, C—H⋯O inter­actions occur along the [100] direction.

## Related literature

For background to the biological activity of *N*-substituted benzamides and their use in synthesis, see: Priya *et al.* (2005 ▶). For related structures and their use in mol­ecular recognition, see: Toda *et al.* (1987 ▶); Saeed *et al.* (2011 ▶, 2012 ▶). For puckering parameters, see Cremer & Pople (1975 ▶). For a description of the Cambridge Structural Database, see: Allen (2002 ▶).
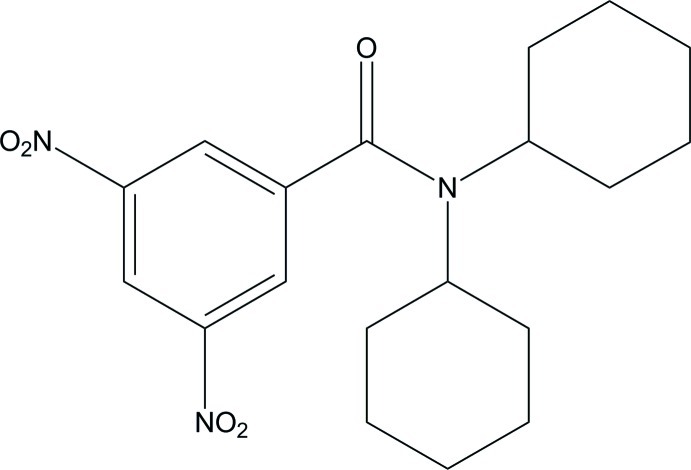


## Experimental

### 

#### Crystal data

C_19_H_25_N_3_O_5_
*M* *_r_* = 375.42
Triclinic, 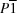

*a* = 6.8187 (7) Å
*b* = 9.7877 (12) Å
*c* = 14.7423 (12) Å
α = 92.512 (8)°
β = 98.898 (8)°
γ = 99.704 (9)°
*V* = 955.67 (17) Å^3^
*Z* = 2
Cu *K*α radiation
μ = 0.79 mm^−1^
*T* = 123 K
0.51 × 0.17 × 0.04 mm

#### Data collection

Agilent Xcalibur Ruby Gemini diffractometer
Absorption correction: multi-scan [*CrysAlis RED* (Agilent, 2011 ▶), based on expressions derived from Clark & Reid (1995 ▶)] *T*_min_ = 0.690, *T*_max_ = 0.969
6078 measured reflections
3811 independent reflections
3144 reflections with *I* > 2σ(*I*)
*R*_int_ = 0.032

#### Refinement

*R*[*F* ^2^ > 2σ(*F* ^2^)] = 0.045
*wR*(*F* ^2^) = 0.132
*S* = 1.03
3811 reflections
244 parameters
H-atom parameters constrained
Δρ_max_ = 0.30 e Å^−3^
Δρ_min_ = −0.23 e Å^−3^

### 

Data collection: *CrysAlis PRO* (Agilent, 2011 ▶); cell refinement: *CrysAlis PRO*; data reduction: *CrysAlis PRO*; program(s) used to solve structure: *SHELXTL* (Sheldrick, 2008 ▶); program(s) used to refine structure: *SHELXTL*; molecular graphics: *SHELXTL*; software used to prepare material for publication: *SHELXTL*.

## Supplementary Material

Crystal structure: contains datablock(s) I, global. DOI: 10.1107/S1600536812038500/nk2179sup1.cif

Structure factors: contains datablock(s) I. DOI: 10.1107/S1600536812038500/nk2179Isup2.hkl

Supplementary material file. DOI: 10.1107/S1600536812038500/nk2179Isup3.cml

Additional supplementary materials:  crystallographic information; 3D view; checkCIF report

## Figures and Tables

**Table 1:** Hydrogen-bond geometry (Å, °)

*D*—H⋯*A*	*D*—H	H⋯*A*	*D*⋯*A*	*D*—H⋯*A*
C4—H4*A*⋯O4^i^	0.95	2.56	3.418 (2)	151
C16—H16*B*⋯O3^ii^	0.99	2.58	3.468 (2)	149

Symmetry codes: (i) 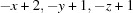; (ii) 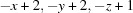.

**Fig. 1.**
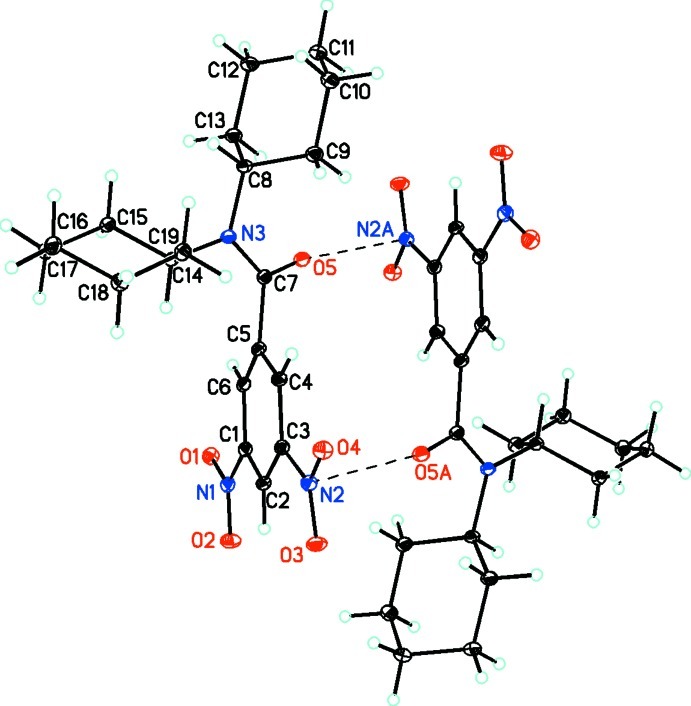
Molecular structure of the title compound with 30% probability displacement ellipsoids. The diagram also shows the association of the molecules into dimeric units through C=O···nitro interamolecular interactions [symmetry code: (A) = 1 - x, 1 - y, 1 - z].

## supplementary crystallographic information

### Comment

The structure of the title compound, (I), was been determined to explore the effect of substituents on the structure of benzanilides (Saeed *et al.*, 2011, 2012). A compound with the same basic skeleton as the title compound has been used in host–guest chemistry to form numerous highly crystalline adducts with a variety of common organic solvents (Toda *et al.*, 1987).

The crystal structure and atom numbering of (I) is shown in Fig. 1. The nitro groups are almost coplanar with the attached benzene ring (dihedral angles between phenyl ring and nitro groups being 1.74 (27) and 6.43 (33)°, respectively). As in the related structure reported recently (Saeed, *et al.*, 2012), the phenyl ring is not coplanar with the amide moiety (dihedral angle between planes C5—C7—N3—O5 and the phenyl ring of 61.90 (5) °). Also analogous with this structure the phenyl ring is twisted out of this plane as indicated by the C4 C5 C7 O5 torsion angle of 112.76 (16) ° (in the previous structure containing two molecules in the symmetric unit these angles were 121.46 (33)° and -119.62 (34)°). The cyclohexyl rings are both in a chair conformation [the puckering parameters (Cremer & Pople, 1975) are Q(2) and φ(2) 0.018 (1) Å and 4.184 (1) ° in C8—C13, 0.025 (1) Å and 305.147 (1) ° in C14—C19). There is a strong intermolecular interaction between the C=O group of the amide moiety and the nitro group of an adjoining molecule (O5···N2 distance of 2.7862 (17) Å) so that these molecules form a dimeric unit. A search of the Cambridge Structural Database (Allen, 2002) for similar intermolecular interactions between carbonyl groups and nitro groups showed that such interactions are not universal. There were over 7000 hits for structures containing both these groups but only 515 contained such interactions with a mean distance of 2.963 Å) and a minimum of 2.73 Å. Thus this interaction in this compound is one of the strongest to be observed. While there are no classic hydrogen bonds found in the crystal, there are weak C—H···O intra- and intermolecular interactions.

### Experimental

To a 250 ml round flask fitted with a condenser was added dicyclohexyl amine (0.01 mol), dichloromethane (15 ml) and triethylamine(0.5 ml) with magnetic stirring. 3,5-dinitrobenzoyl chloride (0.01 mol) was added gradually. The reaction mixture was stirred at room temperature for 1 h and then refluxed for 2 h. The product precipitated as a colorless powder, which was washed three times with water and dichloromethane. Recrystallization from ethyl acetate produced the crystals of the title compound.

### Refinement

The H atoms were placed in their calculated positions with C—H 0.95 and 0.99 Å and refined using the riding model with isotropic displacement parameters set to 1.2 times *U*_eq_ of the parent atoms.

### Crystal data

**Table:**

C_19_H_25_N_3_O_5_	*Z* = 2
*M**_r_* = 375.42	*F*(000) = 400
Triclinic, *P*1	*D*_x_ = 1.305 Mg m^−^^3^
Hall symbol: -P 1	Cu *K*α radiation, λ = 1.54184 Å
*a* = 6.8187 (7) Å	Cell parameters from 2277 reflections
*b* = 9.7877 (12) Å	θ = 3.0–75.2°
*c* = 14.7423 (12) Å	µ = 0.79 mm^−^^1^
α = 92.512 (8)°	*T* = 123 K
β = 98.898 (8)°	`needle-plate`, colorless
γ = 99.704 (9)°	0.51 × 0.17 × 0.04 mm
*V* = 955.67 (17) Å^3^	

### Data collection

**Table:**

Agilent Xcalibur Ruby Gemini diffractometer	3811 independent reflections
Radiation source: Enhance (Cu) X-ray Source	3144 reflections with *I* > 2σ(*I*)
Graphite monochromator	*R*_int_ = 0.032
Detector resolution: 10.5081 pixels mm^-1^	θ_max_ = 75.4°, θ_min_ = 3.0°
ω scans	*h* = −8→7
Absorption correction: multi-scan [*CrysAlis RED* (Agilent, 2011), based on expressions derived from Clark & Reid (1995)]	*k* = −10→12
*T*_min_ = 0.690, *T*_max_ = 0.969	*l* = −18→15
6078 measured reflections	

### Refinement

**Table:**

Refinement on *F*^2^	Primary atom site location: structure-invariant direct methods
Least-squares matrix: full	Secondary atom site location: difference Fourier map
*R*[*F*^2^ > 2σ(*F*^2^)] = 0.045	Hydrogen site location: inferred from neighbouring sites
*wR*(*F*^2^) = 0.132	H-atom parameters constrained
*S* = 1.03	*w* = 1/[σ^2^(*F*_o_^2^) + (0.0695*P*)^2^ + 0.0476*P*] where *P* = (*F*_o_^2^ + 2*F*_c_^2^)/3
3811 reflections	(Δ/σ)_max_ < 0.001
244 parameters	Δρ_max_ = 0.30 e Å^−^^3^
0 restraints	Δρ_min_ = −0.23 e Å^−^^3^

### Special details

**Table:**

Experimental. Absorption correction: CrysAlis RED, (Agilent, 2011) Empirical absorption correction using spherical harmonics, implemented in SCALE3 ABSPACK scaling algorithm. (Clark & Reid, 1995).
Geometry. All e.s.d.'s (except the e.s.d. in the dihedral angle between two l.s. planes) are estimated using the full covariance matrix. The cell e.s.d.'s are taken into account individually in the estimation of e.s.d.'s in distances, angles and torsion angles; correlations between e.s.d.'s in cell parameters are only used when they are defined by crystal symmetry. An approximate (isotropic) treatment of cell e.s.d.'s is used for estimating e.s.d.'s involving l.s. planes.
Refinement. Refinement of *F*^2^ against ALL reflections. The weighted *R*-factor *wR* and goodness of fit *S* are based on *F*^2^, conventional *R*-factors *R* are based on *F*, with *F* set to zero for negative *F*^2^. The threshold expression of *F*^2^ > σ(*F*^2^) is used only for calculating *R*-factors(gt) *etc*. and is not relevant to the choice of reflections for refinement. *R*-factors based on *F*^2^ are statistically about twice as large as those based on *F*, and *R*- factors based on ALL data will be even larger.

### Fractional atomic coordinates and isotropic or equivalent isotropic displacement parameters (Å^2^)

**Table:**

	*x*	*y*	*z*	*U*_iso_*/*U*_eq_	
O1	0.23442 (17)	0.94242 (13)	0.55223 (8)	0.0308 (3)	
O2	0.4677 (2)	0.98065 (14)	0.67312 (8)	0.0362 (3)	
O3	1.03595 (18)	0.75165 (13)	0.69435 (7)	0.0325 (3)	
O4	1.08761 (16)	0.63134 (13)	0.57626 (8)	0.0303 (3)	
O5	0.29440 (16)	0.51429 (12)	0.34083 (7)	0.0274 (3)	
N1	0.3957 (2)	0.92478 (14)	0.59620 (9)	0.0259 (3)	
N2	0.98833 (19)	0.70201 (14)	0.61477 (8)	0.0252 (3)	
N3	0.54384 (18)	0.61099 (13)	0.26430 (8)	0.0220 (3)	
C1	0.5115 (2)	0.83290 (15)	0.55400 (10)	0.0234 (3)	
C2	0.6958 (2)	0.81731 (16)	0.60284 (9)	0.0234 (3)	
H2A	0.7478	0.8646	0.6615	0.028*	
C3	0.7997 (2)	0.72959 (16)	0.56194 (10)	0.0226 (3)	
C4	0.7312 (2)	0.66292 (16)	0.47469 (9)	0.0228 (3)	
H4A	0.8090	0.6051	0.4479	0.027*	
C5	0.5460 (2)	0.68296 (16)	0.42755 (9)	0.0227 (3)	
C6	0.4320 (2)	0.76562 (16)	0.46813 (10)	0.0233 (3)	
H6A	0.3025	0.7759	0.4377	0.028*	
C7	0.4509 (2)	0.59667 (16)	0.33856 (9)	0.0222 (3)	
C8	0.4805 (2)	0.50539 (16)	0.18488 (9)	0.0230 (3)	
H8A	0.5724	0.5316	0.1394	0.028*	
C9	0.5117 (2)	0.36144 (17)	0.21379 (10)	0.0291 (3)	
H9A	0.4270	0.3328	0.2609	0.035*	
H9B	0.6545	0.3654	0.2415	0.035*	
C10	0.4561 (3)	0.25421 (18)	0.13107 (11)	0.0329 (4)	
H10A	0.5498	0.2776	0.0868	0.040*	
H10B	0.4701	0.1610	0.1519	0.040*	
C11	0.2403 (3)	0.25131 (18)	0.08358 (11)	0.0338 (4)	
H11A	0.2087	0.1835	0.0293	0.041*	
H11B	0.1458	0.2211	0.1264	0.041*	
C12	0.2126 (3)	0.39444 (18)	0.05318 (10)	0.0325 (4)	
H12A	0.0709	0.3907	0.0237	0.039*	
H12B	0.3004	0.4216	0.0070	0.039*	
C13	0.2648 (2)	0.50401 (17)	0.13510 (10)	0.0270 (3)	
H13A	0.2535	0.5969	0.1130	0.032*	
H13B	0.1683	0.4825	0.1784	0.032*	
C14	0.7089 (2)	0.72822 (15)	0.25786 (9)	0.0216 (3)	
H14A	0.7252	0.7921	0.3144	0.026*	
C15	0.6558 (2)	0.81222 (16)	0.17448 (9)	0.0236 (3)	
H15A	0.6357	0.7516	0.1171	0.028*	
H15B	0.5283	0.8461	0.1784	0.028*	
C16	0.8253 (2)	0.93638 (17)	0.17186 (10)	0.0275 (3)	
H16A	0.7932	0.9849	0.1154	0.033*	
H16B	0.8334	1.0027	0.2255	0.033*	
C17	1.0291 (2)	0.89085 (18)	0.17335 (11)	0.0294 (3)	
H17A	1.1359	0.9739	0.1764	0.035*	
H17B	1.0269	0.8340	0.1159	0.035*	
C18	1.0770 (2)	0.80630 (17)	0.25612 (10)	0.0276 (3)	
H18A	1.0916	0.8660	0.3136	0.033*	
H18C	1.2067	0.7745	0.2542	0.033*	
C19	0.9104 (2)	0.68002 (16)	0.25653 (10)	0.0251 (3)	
H19C	0.9008	0.6170	0.2010	0.030*	
H19A	0.9432	0.6283	0.3114	0.030*	

### Atomic displacement parameters (Å^2^)

**Table:**

	*U*^11^	*U*^22^	*U*^33^	*U*^12^	*U*^13^	*U*^23^
O1	0.0303 (6)	0.0312 (6)	0.0329 (6)	0.0102 (5)	0.0063 (4)	0.0034 (5)
O2	0.0422 (7)	0.0403 (7)	0.0264 (5)	0.0109 (5)	0.0059 (5)	−0.0091 (5)
O3	0.0332 (6)	0.0371 (6)	0.0230 (5)	0.0058 (5)	−0.0054 (4)	−0.0046 (5)
O4	0.0261 (5)	0.0339 (6)	0.0309 (5)	0.0080 (5)	0.0028 (4)	−0.0002 (5)
O5	0.0273 (5)	0.0304 (6)	0.0222 (5)	−0.0011 (4)	0.0046 (4)	0.0000 (4)
N1	0.0297 (6)	0.0251 (6)	0.0239 (6)	0.0046 (5)	0.0075 (5)	0.0031 (5)
N2	0.0251 (6)	0.0250 (6)	0.0236 (6)	0.0010 (5)	0.0018 (5)	0.0018 (5)
N3	0.0231 (6)	0.0238 (6)	0.0178 (5)	0.0020 (5)	0.0022 (4)	−0.0009 (4)
C1	0.0281 (7)	0.0222 (7)	0.0208 (6)	0.0032 (6)	0.0077 (5)	0.0030 (5)
C2	0.0281 (7)	0.0239 (7)	0.0171 (6)	0.0007 (5)	0.0048 (5)	0.0006 (5)
C3	0.0223 (6)	0.0241 (7)	0.0204 (6)	0.0009 (5)	0.0031 (5)	0.0031 (5)
C4	0.0248 (7)	0.0243 (7)	0.0195 (6)	0.0030 (5)	0.0065 (5)	0.0006 (5)
C5	0.0266 (7)	0.0248 (7)	0.0158 (6)	0.0009 (5)	0.0045 (5)	0.0024 (5)
C6	0.0241 (6)	0.0264 (7)	0.0192 (6)	0.0030 (5)	0.0033 (5)	0.0051 (5)
C7	0.0242 (6)	0.0230 (7)	0.0194 (6)	0.0055 (5)	0.0020 (5)	0.0017 (5)
C8	0.0272 (7)	0.0239 (7)	0.0167 (6)	0.0021 (5)	0.0035 (5)	−0.0013 (5)
C9	0.0344 (8)	0.0282 (8)	0.0234 (7)	0.0087 (6)	−0.0021 (6)	−0.0001 (6)
C10	0.0441 (9)	0.0260 (8)	0.0292 (8)	0.0103 (7)	0.0044 (7)	−0.0024 (6)
C11	0.0447 (9)	0.0268 (8)	0.0249 (7)	0.0002 (7)	−0.0009 (7)	−0.0044 (6)
C12	0.0403 (9)	0.0312 (8)	0.0213 (7)	0.0040 (7)	−0.0050 (6)	−0.0033 (6)
C13	0.0308 (7)	0.0273 (8)	0.0213 (7)	0.0073 (6)	−0.0017 (6)	−0.0021 (6)
C14	0.0242 (6)	0.0230 (7)	0.0169 (6)	0.0026 (5)	0.0032 (5)	0.0006 (5)
C15	0.0267 (7)	0.0250 (7)	0.0191 (6)	0.0053 (5)	0.0027 (5)	0.0021 (5)
C16	0.0323 (7)	0.0251 (7)	0.0245 (7)	0.0027 (6)	0.0045 (6)	0.0043 (6)
C17	0.0288 (7)	0.0309 (8)	0.0271 (7)	−0.0007 (6)	0.0065 (6)	0.0038 (6)
C18	0.0228 (7)	0.0316 (8)	0.0270 (7)	0.0023 (6)	0.0025 (5)	0.0032 (6)
C19	0.0235 (7)	0.0275 (7)	0.0244 (7)	0.0045 (6)	0.0042 (5)	0.0034 (5)

### Geometric parameters (Å, º)

**Table:**

O1—N1	1.2300 (18)	C10—H10A	0.9900
O2—N1	1.2284 (18)	C10—H10B	0.9900
O3—N2	1.2235 (17)	C11—C12	1.522 (2)
O4—N2	1.2253 (18)	C11—H11A	0.9900
O5—C7	1.2299 (19)	C11—H11B	0.9900
N1—C1	1.470 (2)	C12—C13	1.536 (2)
N2—C3	1.4696 (19)	C12—H12A	0.9900
N3—C7	1.3486 (19)	C12—H12B	0.9900
N3—C14	1.4831 (18)	C13—H13A	0.9900
N3—C8	1.4874 (17)	C13—H13B	0.9900
C1—C2	1.384 (2)	C14—C19	1.528 (2)
C1—C6	1.387 (2)	C14—C15	1.5392 (19)
C2—C3	1.376 (2)	C14—H14A	1.0000
C2—H2A	0.9500	C15—C16	1.536 (2)
C3—C4	1.391 (2)	C15—H15A	0.9900
C4—C5	1.393 (2)	C15—H15B	0.9900
C4—H4A	0.9500	C16—C17	1.526 (2)
C5—C6	1.389 (2)	C16—H16A	0.9900
C5—C7	1.5199 (19)	C16—H16B	0.9900
C6—H6A	0.9500	C17—C18	1.529 (2)
C8—C9	1.529 (2)	C17—H17A	0.9900
C8—C13	1.536 (2)	C17—H17B	0.9900
C8—H8A	1.0000	C18—C19	1.532 (2)
C9—C10	1.531 (2)	C18—H18A	0.9900
C9—H9A	0.9900	C18—H18C	0.9900
C9—H9B	0.9900	C19—H19C	0.9900
C10—C11	1.523 (2)	C19—H19A	0.9900
			
O2—N1—O1	123.96 (14)	C12—C11—H11B	109.5
O2—N1—C1	117.72 (13)	C10—C11—H11B	109.5
O1—N1—C1	118.31 (13)	H11A—C11—H11B	108.1
O3—N2—O4	124.38 (13)	C11—C12—C13	111.33 (13)
O3—N2—C3	117.65 (13)	C11—C12—H12A	109.4
O4—N2—C3	117.97 (12)	C13—C12—H12A	109.4
C7—N3—C14	122.84 (12)	C11—C12—H12B	109.4
C7—N3—C8	119.34 (12)	C13—C12—H12B	109.4
C14—N3—C8	117.82 (11)	H12A—C12—H12B	108.0
C2—C1—C6	123.08 (15)	C8—C13—C12	110.00 (13)
C2—C1—N1	117.81 (13)	C8—C13—H13A	109.7
C6—C1—N1	119.12 (14)	C12—C13—H13A	109.7
C3—C2—C1	116.45 (13)	C8—C13—H13B	109.7
C3—C2—H2A	121.8	C12—C13—H13B	109.7
C1—C2—H2A	121.8	H13A—C13—H13B	108.2
C2—C3—C4	123.15 (14)	N3—C14—C19	112.10 (12)
C2—C3—N2	117.98 (13)	N3—C14—C15	111.61 (11)
C4—C3—N2	118.84 (14)	C19—C14—C15	111.05 (12)
C3—C4—C5	118.39 (14)	N3—C14—H14A	107.3
C3—C4—H4A	120.8	C19—C14—H14A	107.3
C5—C4—H4A	120.8	C15—C14—H14A	107.3
C6—C5—C4	120.26 (13)	C16—C15—C14	110.47 (12)
C6—C5—C7	118.74 (13)	C16—C15—H15A	109.6
C4—C5—C7	120.25 (14)	C14—C15—H15A	109.6
C1—C6—C5	118.57 (14)	C16—C15—H15B	109.6
C1—C6—H6A	120.7	C14—C15—H15B	109.6
C5—C6—H6A	120.7	H15A—C15—H15B	108.1
O5—C7—N3	124.41 (13)	C17—C16—C15	111.74 (13)
O5—C7—C5	116.40 (12)	C17—C16—H16A	109.3
N3—C7—C5	119.15 (12)	C15—C16—H16A	109.3
N3—C8—C9	110.96 (11)	C17—C16—H16B	109.3
N3—C8—C13	113.26 (12)	C15—C16—H16B	109.3
C9—C8—C13	111.99 (13)	H16A—C16—H16B	107.9
N3—C8—H8A	106.7	C16—C17—C18	110.80 (13)
C9—C8—H8A	106.7	C16—C17—H17A	109.5
C13—C8—H8A	106.7	C18—C17—H17A	109.5
C8—C9—C10	110.91 (12)	C16—C17—H17B	109.5
C8—C9—H9A	109.5	C18—C17—H17B	109.5
C10—C9—H9A	109.5	H17A—C17—H17B	108.1
C8—C9—H9B	109.5	C17—C18—C19	111.27 (12)
C10—C9—H9B	109.5	C17—C18—H18A	109.4
H9A—C9—H9B	108.0	C19—C18—H18A	109.4
C11—C10—C9	110.78 (14)	C17—C18—H18C	109.4
C11—C10—H10A	109.5	C19—C18—H18C	109.4
C9—C10—H10A	109.5	H18A—C18—H18C	108.0
C11—C10—H10B	109.5	C14—C19—C18	109.64 (13)
C9—C10—H10B	109.5	C14—C19—H19C	109.7
H10A—C10—H10B	108.1	C18—C19—H19C	109.7
C12—C11—C10	110.67 (14)	C14—C19—H19A	109.7
C12—C11—H11A	109.5	C18—C19—H19A	109.7
C10—C11—H11A	109.5	H19C—C19—H19A	108.2
			
O2—N1—C1—C2	−1.2 (2)	C6—C5—C7—N3	124.80 (15)
O1—N1—C1—C2	177.97 (13)	C4—C5—C7—N3	−65.11 (19)
O2—N1—C1—C6	178.46 (14)	C7—N3—C8—C9	−60.72 (17)
O1—N1—C1—C6	−2.4 (2)	C14—N3—C8—C9	119.11 (14)
C6—C1—C2—C3	−0.3 (2)	C7—N3—C8—C13	66.24 (17)
N1—C1—C2—C3	179.38 (12)	C14—N3—C8—C13	−113.94 (14)
C1—C2—C3—C4	2.5 (2)	N3—C8—C9—C10	−177.25 (12)
C1—C2—C3—N2	−175.35 (13)	C13—C8—C9—C10	55.10 (17)
O3—N2—C3—C2	4.5 (2)	C8—C9—C10—C11	−56.00 (18)
O4—N2—C3—C2	−175.30 (13)	C9—C10—C11—C12	57.49 (18)
O3—N2—C3—C4	−173.47 (13)	C10—C11—C12—C13	−57.87 (19)
O4—N2—C3—C4	6.7 (2)	N3—C8—C13—C12	178.94 (12)
C2—C3—C4—C5	−1.8 (2)	C9—C8—C13—C12	−54.65 (17)
N2—C3—C4—C5	176.03 (13)	C11—C12—C13—C8	55.91 (18)
C3—C4—C5—C6	−1.2 (2)	C7—N3—C14—C19	113.34 (15)
C3—C4—C5—C7	−171.14 (13)	C8—N3—C14—C19	−66.48 (16)
C2—C1—C6—C5	−2.6 (2)	C7—N3—C14—C15	−121.37 (14)
N1—C1—C6—C5	177.76 (13)	C8—N3—C14—C15	58.81 (16)
C4—C5—C6—C1	3.3 (2)	N3—C14—C15—C16	177.61 (12)
C7—C5—C6—C1	173.39 (13)	C19—C14—C15—C16	−56.51 (16)
C14—N3—C7—O5	168.10 (14)	C14—C15—C16—C17	54.76 (16)
C8—N3—C7—O5	−12.1 (2)	C15—C16—C17—C18	−54.88 (17)
C14—N3—C7—C5	−14.2 (2)	C16—C17—C18—C19	56.63 (18)
C8—N3—C7—C5	165.61 (13)	N3—C14—C19—C18	−176.37 (11)
C6—C5—C7—O5	−57.32 (19)	C15—C14—C19—C18	58.03 (15)
C4—C5—C7—O5	112.77 (16)	C17—C18—C19—C14	−58.10 (17)

### Hydrogen-bond geometry (Å, º)

**Table:**

*D*—H···*A*	*D*—H	H···*A*	*D*···*A*	*D*—H···*A*
C9—H9*A*···O5	0.99	2.46	3.050 (2)	118
C13—H13*B*···O5	0.99	2.40	3.0043 (18)	119
C4—H4*A*···O4^i^	0.95	2.56	3.418 (2)	151
C16—H16*B*···O3^ii^	0.99	2.58	3.468 (2)	149

Symmetry codes: (i) −*x*+2, −*y*+1, −*z*+1; (ii) −*x*+2, −*y*+2, −*z*+1.
